# Genotypes and clinical intervention of patients with neurofibromatosis type 1 associated dystrophic scoliosis

**DOI:** 10.3389/fped.2022.918136

**Published:** 2022-08-18

**Authors:** Haichong Li, Wenyan Zhang, Ziming Yao, Ruolan Guo, Chanjuan Hao, Xuejun Zhang

**Affiliations:** ^1^Department of Orthopedics, Beijing Children's Hospital, Capital Medical University, National Center for Children's Health, Beijing, China; ^2^Beijing Key Laboratory for Genetics of Birth Defects, Beijing Pediatric Research Institute, Beijing, China; ^3^MOE Key Laboratory of Major Diseases in Children, Beijing, China; ^4^Genetics and Birth Defects Control Center, Beijing Children's Hospital, Capital Medical University, National Center for Children's Health, Beijing, China; ^5^Henan Key Laboratory of Pediatric Inherited & Metabolic Diseases, Henan Children's Hospital, Zhengzhou Hospital of Beijing Children's Hospital, Zhengzhou, China

**Keywords:** NF1, exome sequencing, dystrophic scoliosis, growing-rod surgery, posterior spinal fusion, mutation detection rate

## Abstract

**Objective:**

To analyze the genotypic characteristics of patients with neurofibromatosis type 1 (NF1) associated dystrophic scoliosis and to summarize the outcomes of the surgical treatment of these patients.

**Methods:**

Exome sequencing (ES) combined with multiplex ligation-dependent probe amplification (MLPA) was used for genotypic identification. All patients underwent surgical treatments for spinal deformities, and the outcomes of the surgery was summarized by analyzing the clinical and imaging parameters before and after the surgery.

**Results:**

Fourteen patients (six males and eight females) were clinically diagnosed as NF1 associated dystrophic scoliosis with common symptoms including café-au-lait spots, paravertebral tumors, and dystrophic scoliosis. *NF1* mutations were detected in 12 (85.7%) patients, including four nonsense mutations, three splicing mutations, three frameshift mutations, and two exon deletions. The first surgical procedure included growing-rod surgery in 10 patients and posterior spinal fusion in four patients. The follow-up duration was 2.3 years (1.0–10.3 years), and the Cobb angle of the main curve improved from 61.5° (30°-125°) pre-operatively to 14.5° (0°-42°) at the last follow-up, with an average correction rate of 74.0% (44–100%). Instrumentation-related complications occurred in four patients during the follow-up period.

**Conclusions:**

In patients with dystrophic scoliosis who met the clinical diagnostic criteria for NF1, the mutation detection rate of ES combined with MLPA was 85.7%. There was no mutation hotspot in *NF1* gene, molecular diagnosis could offer information about genetic counseling, prenatal diagnosis and eugenics. Surgical treatment according to patient's age and severity could effectively correct the spinal deformities.

## Introduction

Neurofibromatosis type 1 (NF1) is an autosomal dominant genetic disorder, with an incidence of ~1/3,000. The pathogenic gene *NF1*, is located in 17q11.2, with 60 exons and a total length of ~350 kb. *NF1* mutations lead to the dysfunction of the NF1 protein, resulting in café-au-lait spots, plexus neurofibroma, optic glioma, skeletal system disorders, and other manifestations. NF1 associated dystrophic scoliosis is the most common skeletal system disorder, with an incidence of 30% (1). The imaging manifestations of spinal deformity include vertebral scalloping, rib penciling, elongated and attenuated pedicles, and a widened spinal canal and foramen (2, 3).

Most cases of NF1 associated dystrophic scoliosis are progressive, and conservative treatment is usually ineffective. Considering the potential for spinal growth, growing-rod surgery is typically performed in young patients. For elderly patients, posterior spinal fusion is usually used to correct deformities and to control their progression (4). Due to severity and complexity of spinal deformities in patients with NF1, surgical treatment is difficult, and the incidence of complications, such as displacement, breakage, and loosening of instrumentation, as well as deformity progression, could be more than 50% (5, 6).

Previous studies focused on either genotypic analysis of patients with NF1 (7, 8) or surgical effects and complications of surgery for NF1 associated dystrophic scoliosis (9, 10), while hardly any reports comprehensively studied the genotypic characteristics of patients with NF1 associated dystrophic scoliosis and their respective effects of clinical intervention.

In our study, we recruited patients clinically diagnosed with NF1 associated dystrophic scoliosis in Beijing Children's hospital and investigated their genotypic characteristics using exome sequencing (ES) combined with multiplex ligation-dependent probe amplification (MLPA). All of patients accepted surgical treatments to control the progression of spinal deformities. Their clinical and imaging parameters before and after surgical treatments were analyzed to summarize the effects of clinical interventions.

## Materials and methods

### Subjects

We recruited patients from Department of Orthopedics of Beijing Children's Hospital from May 2010 to December 2021, and collected the complete clinical and imaging data. The patients were diagnosed as NF1 associated dystrophic scoliosis by at least two independent surgeons. The NF1 clinical diagnostic criteria included: (1) six or more café-au-lait spots ≥5 mm in diameter before puberty or ≥1.5 mm in diameter after puberty; (2) axillary or inguinal skinfold freckling; (3) two or more dermal neurofibromas or one plexiform neurofibroma; (4) two or more iris hamartomas (Lisch nodules); (5) An optic pathway glioma; (6) distinctive long bone dysplasia involving the sphenoid wing or thinning of the long bone cortex with or without pseudarthrosis; and (7) A first-degree relative with neurofibromatosis type 1. NF1 can be diagnosed if an individual presents with 2 or more of these features (11).

A total of 14 patients with the clinical diagnosis of NF1 associated dystrophic scoliosis were included. Patients and/or their guardians were informed about this study, and signed the informed consent. This study was approved by the Ethics Committee of Beijing Children's Hospital, Capital Medical University (BCH, Approval No.2022-E-083-R).

### Clinical data

Clinical data, including sex, age, past medical history, and NF1 family history were recorded. Physical examination involved checks for café-au-lait spots, plexus neurofibroma, axillary or inguinal freckles, optic glioma, Lisch nodules (iris hamartoma), and skeletal manifestations. Before surgeries, the patients underwent X-ray, computed tomography (CT), and magnetic resonance imaging (MRI) of the whole spine. The location of the main curve and Cobb angle were measured using an X-ray film (12). The number of malformed vertebrae was determined and recorded according to the CT images of the whole spine. The malformed vertebrae were defined as a spinal curve in the presence of vertebral scalloping, rib penciling, elongated and attenuated pedicles, and a widened spinal canal and foramen (2, 3). The presence of paravertebral or intraspinal tumors was determined using MRI.

### Genetic testing

#### Exome sequencing (ES) and variants analysis

DNA was isolated from peripheral blood samples of patients using the Gentra Puregene Blood Kit (QIAGEN, Hilden, Germany). Exon capture was performed using SureSelect Human All Exon Kit (Agilent Technologies, Santa Clara, America). Target regions were sequenced using NovaSeq (Illumina, San Diego, USA) and compared with the GRCh37/hg19 human reference sequence. Sequencing depth of >100X was employed. Single nucleotide variants (SNVs) and insertion-deletions (indels) were annotated and filtered by TGex (https://fa.shanyint.com). Variants with a frequency over 1% in the databases of gnomAD, ESP or 1000G were excluded. The main disease reference databases included HGMD Professional, Clinvar, OMIM and Malacards. The pathogenicity of the missense variants found in patient was evaluated by listing bioinformatic tools: PolyPhen-2, PROVEAN and MutationTaster, that of splicing variants were predicted by GeneSplicer, MaxEntScan and NetGene2. Variants were classified following the American College of Medical Genetics and Genomics and the Association for Molecular Pathology (ACMG/AMP) interpretation standards and guidelines (13). Putative pathogenic variants detected by ES were confirmed by Sanger sequencing for patients and/or their parents if available. Additional copy number variants (CNVs) based on exome sequencing data were generated using the CNV detection program CNVkit, the samples of the same batch were used as controls.

#### Multiplex ligation-dependent probe amplification (MLPA) and copy number variation (CNV) analysis

**Figure 1:**
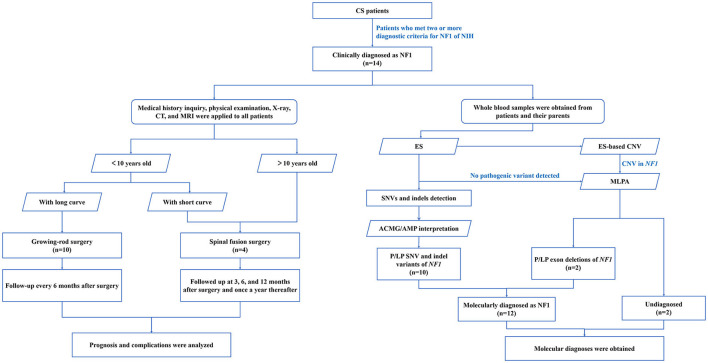
Flow diagram of NF1 associated dystrophic scoliosis patients who were enrolled and received genetic testing. A total of 14 patients with the clinical diagnosis of NF1 associated dystrophic scoliosis were included. Medical history inquiry, physical examination, X-ray, CT, and MRI were applied to all patients to design the operation plan. Young patients with long curve accepted growing-rod surgery, young patients with short curve and older patients accepted spinal fusion surgery. After regular follow-up, prognosis and complications were analyzed. Whole blood samples were obtained from patients and their parents in parallel. Combination of ES and MLPA found 12 *NF1* variants from 12 patients. ES, exome sequencing; MLPA, multiplex ligation-dependent probe amplification; SNV, single nucleotide variants; indels, insertion-deletions; P, pathogenic.

For patients whose ES results suggested ES-based exon deletion and/or didn't found pathogenic/likely pathogenic variants in *NF1*, they were further identified by MLPA (Figure 1). All of *NF1* exons were captured and amplified using SALSA MLPA Probemix P081 NF1 Mix 1 and P082 NF1 Mix 2 (MRC Holland, Netherlands). Electrophoresis of the amplified products was performed using an ABI 3500 gene analyzer (Thermo Scientific, USA), and the results were analyzed using Coffalyser. Net (MRC Holland, Netherlands). The final ratio (FR) of each individual reference probe in the patient samples should be between 0.80 and 1.20, exons with FR between 0.40 and 0.65 indicated heterozygous deletion, while exons with FR between 1.30 and 1.65 indicated heterozygous duplication.

### Surgical treatment

#### Growing-rod surgery

The upper and lower anchor points were located using fluoroscopy during the operation, with two to three vertebrae at each end. Two small longitudinal posterior incisions were made to expose the posterior structure of the corresponding vertebrae, and pedicle screws were inserted. Each set of screws was connected by pre-contoured submuscular rods tunneled to the overlap point, where they were linked with side-by-side “domino” implants. The screws and domino implants were tightened after distraction. Through a small incision over the domino, lengthening was performed at ~6–12 months intervals.

#### Posterior spinal fusion

A posterior midline incision was made to expose the spine. The bilateral pedicle screws were inserted, according to the pre-operative plan. At the levels to be fused, multisegment posterior column osteotomies were performed, the vertebral lamina was decorticated, and a bone graft was placed along the side of the vertebrae.

### Follow-up

Patients with growing rods were followed up every 6 months after surgery, and the lengthening interval was 6–12 months. Patients who underwent posterior spinal fusion were followed up at 3, 6, and 12 months after surgery and once a year thereafter. Standing anteroposterior and lateral radiographs of the entire spine were evaluated at each follow-up.

### Statistical analysis

SPSS18.0 (SPSS Inc., Chicago, IL, USA) was used for statistical analysis. Continuous variables were represented as median (minimum, maximum), and variables were classified by the number of cases (percentage). The Cobb angle correction rate was calculated as [(pre-operative Cobb–post-operative Cobb)/pre-operative Cobb] ×100%. The Wilcoxon test was used to compare the Cobb angle before and after surgery, and statistical significance was set at *P* < 0.05.

## Results

### Clinical data

Fourteen patients diagnosed with NF1 associated dystrophic scoliosis were reviewed (Figure 1), including 6 males and 8 females. The mean age at the initial surgery was 6 years (3–14 years). Among the 14 patients, three patients had reported family history of NF1: Café-au-lait spots and axillary or inguinal skinfold freckling were present at mother of patient 3 and patient 14. Café-au-lait spots and dermal neurofibromas were present at father and grandmother of patient 13 (Table 1).

**Table 1:** Clinical information of 14 patients with NF1 associated dystrophic scoliosis.

**Patient ID**	**Sex**	**Age**	**First operation**	**Apical vertebrae**	**Preoperative Cobb**	**Last follow-up Cobb**	**Last follow-up correction rate (%)**	**Complication**	**Family history**	**Other symptoms**
1	F	5 years 2 months	GR	T5	125	42	66.4	Rod breakage	None	Pulmonary function limitation
2	M	3 years 2 months	GR	T8	63	35	44.4	-	None	None
3	F	9 years 8 months	GR	T9	60	15	75.0	-	Mother	T6-T8 meningocele
4	F	6 years 1 months	GR	T5	94	9	90.4	-	None	None
5	F	13 years 9 months	SF	T4	82	5	93.9	Curve progression	None	None
6	M	8 years 7 months	SF	T9	30	0	100.0	-	None	Plexiform neurofibroma
7	F	6 years 1 months	GR	T7	61	24	60.7	-	None	None
8	F	3 years 4 months	GR	T4	90	30	66.7	Screw displacement	None	Pulmonary function limitation
9	M	5 years 6 months	GR	T9	62	14	77.4	-	None	Plexiform neurofibroma
10	F	3 years 11 months	GR	T11	40	10	75.0	-	None	Pulmonary function limitation
11	M	4 years 2 months	GR	T6	35	10	71.4	Adding-on phenomenon	None	Pulmonary function limitation
12	M	9 years 7 months	GR	T9	63	17	73.0	-	None	Plexiform neurofibroma
13	F	9 years 7 months	SF	L1	55	25	54.5	-	Father and grandmother	Unequal length of the lower extremities, intraspinal neurofibroma
14	M	13 years 4 months	SF	L2	49	5	89.8	-	Mother	Pulmonary function limitation

Café-au-lait spots and axillary or inguinal skinfold freckling were present at mother of patient 3 and patient 14. Café-au-lait spots and dermal neurofibromas were present at father and grandmother of patient 13. GR, Growing-rod surgery; SF, Spinal fusion surgery.

Common symptoms of patients included café-au-lait spots, paraspinal tumors, and dystrophic scoliosis. Café-au-lait spots were present at birth, and the age of dystrophic scoliosis onset varied, with a median age of 4 years (2–13 years). Other symptoms included pulmonary function limitation (*n* = 5), plexiform neurofibroma (*n* = 3), T6-T8 meningocele (*n* = 1), unequal length of the lower extremities (*n* = 1), and intraspinal neurofibroma (*n* = 1). T6-T8 meningocele, intraspinal neurofibroma, and complications of unequal length of both lower limbs were treated before the spinal surgery (Table 1).

### Genetic testing

**Figure 2:**
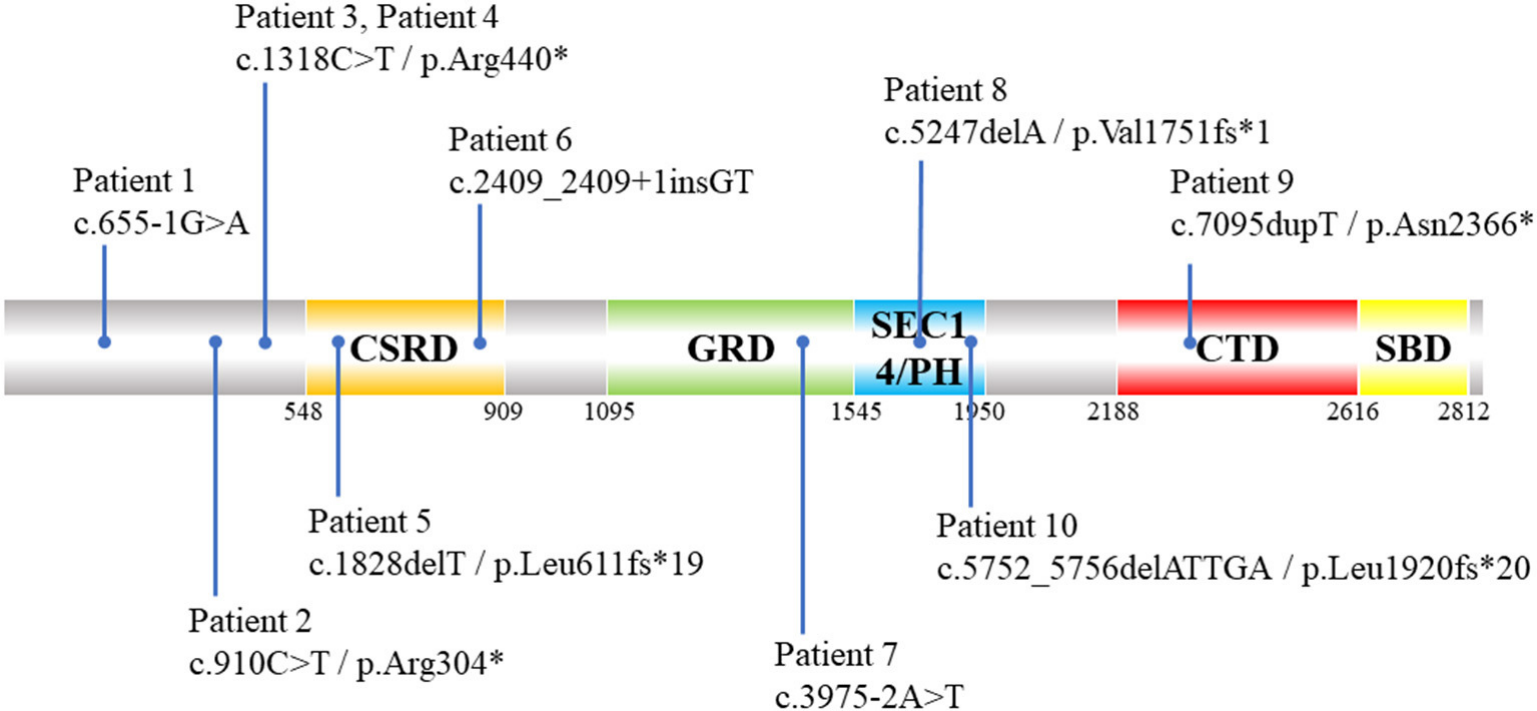
Schematic diagram of distribution of 10 *NF1* pathogenic SNVs and indels variants identified by ES in NF1 associated dystrophic scoliosis patients. CSRD, Cysteine-Serine-rich domain; GRD, GTPase-activating protein-related domain; SEC14/PH, SEC14 domain and pleckstrin homology (PH) domain; CTD, Carboxy-terminal domain; SBD, Syndecan-binding domain; ES, exome sequencing; SNV, single nucleotide variants; indels, insertion-deletions.*means amino acid turns into termination codon and translation is stopped.

The results of ES combined with MLPA showed that 12 patients had *NF1* pathogenic variants (Table 2), with a diagnostic rate of 85.7%. *NF1* mutations included nonsense (*n* = 4), splicing (*n* = 3), frameshift (*n* = 3), and exon deletions (*n* = 2). Five of them were firstly reported (Supplementary Table 1). Among these patients with detected *NF1* variants, their clinical phenotypes and disease progression were not significantly related with their genotypes (Figure 2). In addition, two patients (patients 13 and 14) did not detect any *NF1* mutations in exon regions.

**Table 2:** Variation description of the 12 patients with NF1 associated dystrophic scoliosis.

**Patient ID**	**Detection method**	**Gene**	**Variants type**	**Variants**	**Zygosity**	**Inheritance pattern**	**Firstly reported in literature (No. PMID)**	**Classification**
1	ES	*NF1*	Splicing	c.655-1G>A	Heterozygous	N.A.	8957181	P
2	ES	*NF1*	Nonsense	c.910C>T / p.Arg304^*^	Heterozygous	N.A.	18484666	P
3	ES	*NF1*	Nonsense	c.1318C>T / p.Arg440^*^	Heterozygous	N.A.	18484666	P
4	ES	*NF1*	Nonsense	c.1318C>T / p.Arg440^*^	Heterozygous	N.A.	18484666	P
5	ES	*NF1*	Indel	c.1828delT / p.Leu611fs^*^19	Heterozygous	N.A.	Novel	P
6	ES	*NF1*	Indel	c.2409_2409+1insGT	Heterozygous	N.A.	Novel	P
7	ES	*NF1*	Splicing	c.3975-2A>T	Heterozygous	N.A.	34418705	P
8	ES	*NF1*	Indel	c.5247delA / p.Val1751fs^*^1	Heterozygous	N.A.	Novel	P
9	ES	*NF1*	Indel	c.7095dupT / p.Asn2366^*^	Heterozygous	*De novo*	Novel	P
10	ES	*NF1*	Indel	c.5752_5756delATTGA / p.Leu1920fs^*^20	Heterozygous	*De novo*	Novel	P
11	ES+MLPA	*NF1*	Deletion	exon 15-16 del	Heterozygous	N.A.	16786508	P
12	ES+MLPA^*^	*NF1*	Deletion	exon 1-58 del	Heterozygous	*De novo*	30290804	P

All of patients accepted proband-only ES. Patient 11 and 14 accepted proband-only MLPA.

* Parents of Patient 12 and 13 also accepted MLPA. ES, exome sequencing; MLPA, multiplex ligation-dependent probe amplification; indel, insertion-deletion; N.A., not available; P, pathogenic.

### Imaging evaluation and surgical outcome

Pre-operative imaging revealed paraspinal tumors and vertebral dystrophy in all 14 patients. Dystrophic vertebrae showed wedge-shaped or scallop-like changes, and the number of median deformed vertebrae was 3 (2–7). The apical vertebrae were located in the thoracic vertebrae in 12 cases and in the lumbar vertebrae in two cases. The first surgery included growing-rod surgery in 10 cases and posterior spinal fusion in four cases. The median operative time was 200 min (90–700 min) and the median intraoperative blood loss was 425 mL (140–2,300 mL). The pre-operative median Cobb angle of the main curve was 61.5° (30°-125°) and the postoperative Cobb angle was 25.5° (0°-55°). The immediate correction rate was 60.0% (25–100%), and the postoperative Cobb angle was significantly lower than that before surgery (*Z* = *3.297, P *<*0.001*). At the last follow-up, the Cobb angle of the main curve was 14.5° (0°-42.0°) and the correction rate was 74.0% (44–100%) (Table 1).

### Follow up

The duration of follow-up was 1–10 years, with a median term of 2.3 years. Among the 10 patients who underwent growing-rod surgery, two patients completed the definitive fusion. Postoperative instrumentation-related complications occurred in 4 cases (28.6%). Among the patients who underwent growing-rod surgery, screw displacement, rod breakage, and adding-on phenomenon occurred in one patient each, respectively. Curve progression occurred in one patient who underwent posterior spinal fusion surgery.

**Figure 3:**
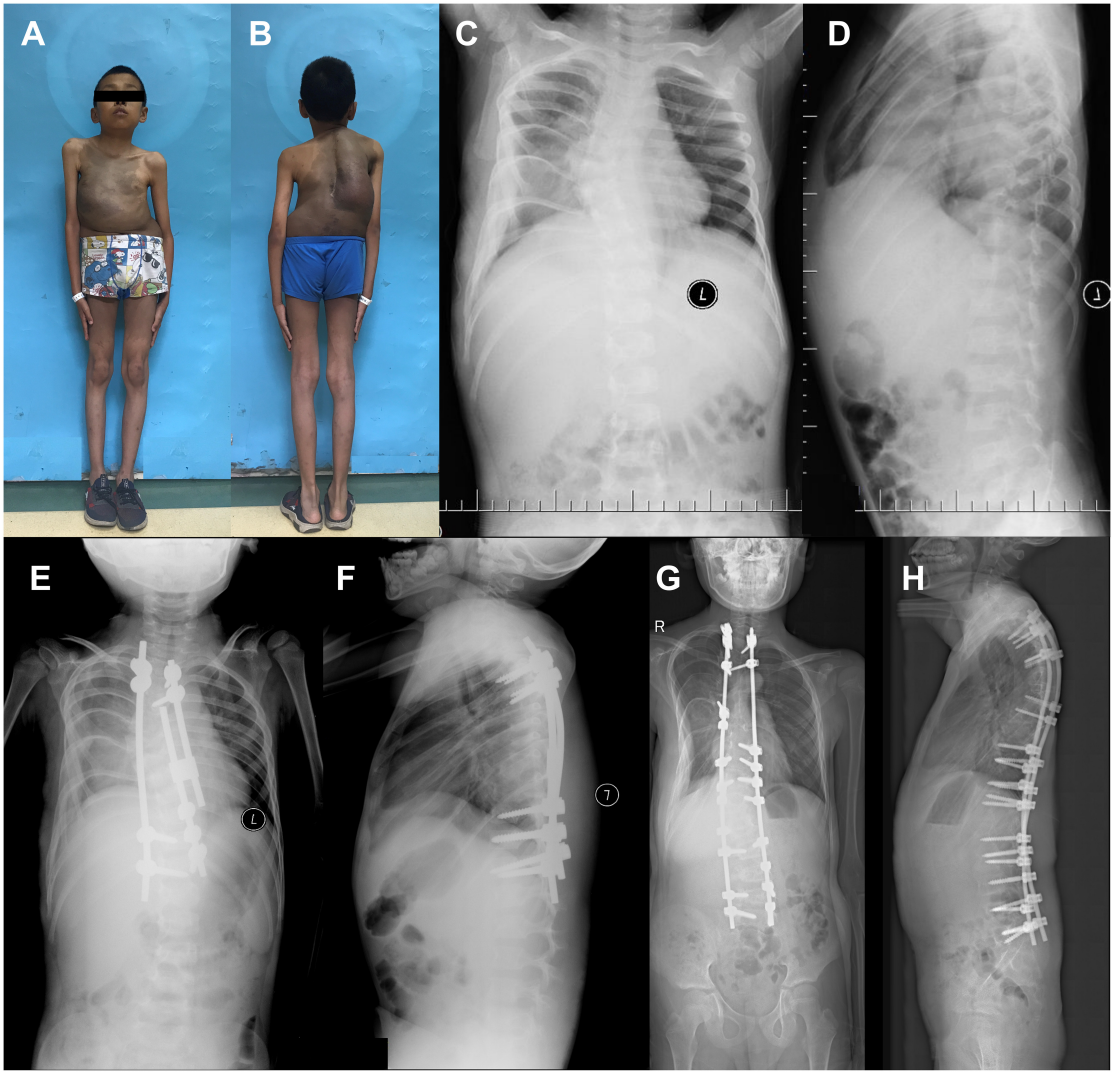
Surgical treatment of 4-year-old NF1 associated dystrophic scoliosis patient 11. **(A,B)** Patient 11 had large café-au-lait spots on the trunk, scattered café-au-lait spots on the limb, and scoliosis. **(C,D)** X-ray of spine showed scoliosis with apical vertebrae located in T6 causing 35.0° Cobb angle, dystrophic changes at T5-T7 vertebrae when patient was 4 years old. **(E,F)** After surgery, X-ray showed that Cobb angle was 10.0°. **(G,H)** After definitive fusion, patient's Cobb angle was 10.0° at 12 years and 7 months old. T, thoracic vertebra.

Two years after the definitive spinal fusion, retroperitoneal rhabdomyosarcoma was observed in Patient 11 (Figure 3; Patient 11 in Table 1), and led to the death of the patient, 1 year later.

## Discussion

NF1 spinal deformity is generally classified into two categories: non-dystrophic and dystrophic, based on the imaging evidence of skeletal dystrophy such as vertebral scalloping, rib penciling, elongated and attenuated pedicles, and a widened spinal canal and foramen (2, 3). Pre-operation imaging revealed skeletal dystrophy in all of 14 patients, in which the *NF1* mutation detection rate of ES combined with MLPA was 85.7%. Our cohort showed that there was no mutation hotspot in *NF1* gene and no clear genotype-phenotype correlation, but all of *NF1* variants were null variants. Surgical treatment according to patients' age and severity could effectively correct the spinal deformities.

Patients without typical clinical manifestations require genetic testing to confirm the diagnosis of NF1. In our study, 12 of 14 clinically diagnosed as NF1 patients were identified as *NF1* null variants by the combination of ES and MLPA, including four nonsense variants, three splicing variants, three frameshift variants, and two exon deletions. All of the variants could reduce mature NF1 protein, leading to neurofibromatosis around the vertebrae, and further scoliosis. In our study, no significant genotype-phenotype correlation was observed to date, consistent with previous reports (7, 14). The combination of ES and MLPA could detect SNVs, indels, and exon-deletions of *NF1*, which could cover the majority of *NF1* mutations (15, 16). We molecularly diagnosed 85.7% of patients who met the clinical criteria of NF1, which highlighted the necessity of the application of ES and MLPA in clinical practice. Among them, SNVs and indels of *NF1* took proportion of 71.4% (10/14) of NF1 patients, so ES should be prior to MLPA for economic efficiency. However, ES and MLPA testing for peripheral blood sample cannot uncover etiology of all NF1 patients, germline mutations located in the *NF1* introns or somatic *NF1* mutations could also lead to NF1 and its inheritance between generations (15, 16). For undiagnosed patients, we will further perform RNA-seq to investigate the variants of *NF1* intron region, or take skin tissues for molecular diagnosis to analyze somatic variants (23).

The surgical treatment of NF1 associated dystrophic scoliosis is relatively difficult for surgeons. First, spinal deformities caused by *NF1* mutations are usually severe and require surgical treatment at a young age. At this age, patients possess low spinal maturity and the pedicle is deformed, leading to a greater difficulty in precise pedicle screw placement (9, 17). Second, *NF1* mutations cause neurofibroma protein deficiency, bone mineralization disorders, and bone strength reduction, resulting in insufficient holding force for internal fixation (17–20). Third, *NF1* mutations lead to a decrease in osteoblast function, which in turn results in a notable decline in the effect of vertebral fusion and an increase in pseudarthrosis (19, 21). In addition, involvement of multiple organs can occur in patients with NF1, leading to complications such as intracranial tumors, intracranial vascular malformations, renal hypertension, cardiopulmonary dysfunction, anisotropy of the cerebromedullary tube, intraspinal tumors, and intervertebral foramen tumors. All these factors can compromise the safety of the surgery. In our study, Patient 3 had complications with T6-T8 meningocele while Patient 13 had intraspinal tumor complications (Table 1). To increase the safety of spinal surgery, T6-T8 meningocele and intraspinal tumors were treated, before performing the spinal surgery.

Surgical management, including growing-rod surgery and spinal fusion surgery, has been recommended to prevent the progression of NF1 associated dystrophic scoliosis. Growing-rod surgery aims to allow truncal growth while maintaining the correction and is necessary for young patients with a long curve (17). Spinal fusion surgery can be performed in patients with a short and sharp curve or >10-year-old patients with a long curve (4). Dystrophic changes in the vertebrae lead to rapid progression of spinal deformities, and early fusion can be considered for patients with frequent instrumentation-related complications following the growing-rod surgery. Jain et al. reported that the growing-rod surgery corrected the early onset scoliosis rate in patients with NF1 from 74° to 36° (51% correction) (9). Wang et al. reported that the rate of correction of scoliosis with posterior pedicle screw fixation from 83.2° to 27.6° (67% correction) (22). In our study, the initial surgery included growing-rod surgery in 10 cases and spinal fusion surgery in four cases. The mean correction rate was 60.0% (25–100%) after the operation. At the last follow-up, the mean correction rate was 74.0% (44–100%), suggesting that both growing-rod surgery and spinal fusion surgery were effective for the correction and control of NF1 associated dystrophic scoliosis. Due to severity and complexity of spinal deformities in patients with NF1, surgical treatment is difficult with high incidence of complications. In our study, the frequency of postoperative instrumentation-related complication was 28.6%, after timely revision, these complications were well-controlled. The choice of surgical method should comprehensively consider the age of the patients, development of the spine, and severity of deformity.

In conclusion, in patients with NF1 associated dystrophic scoliosis who met NIH diagnostic criteria, the *NF1* mutation detection rate of ES combined with MLPA was 85.7%, no mutation hotspot in *NF1* gene was found. The phenotype, the severity and progression of scoliosis in NF1 patients were not significantly related with their genotypes, but with the position of neurofibromatosis, so did surgical treatment options and post-operational prognosis. Both growing-rod and spinal fusion can correct the deformity and control scoliosis progression when applied at proper age. The molecular diagnose could offer information about genetic counseling, prenatal diagnosis and eugenics, surgical treatment and long-term prognosis prediction required evaluation from experienced orthopedists.

## Data availability statement

The data presented in the study are deposited in the gsa-human [Genome Sequence Archive for Human (https://ngdc.cncb.ac.cn/gsa-human/)] repository, accession number PRJCA010950.

## Ethics statement

The studies involving human participants were reviewed and approved by this retrospective study of the Chinese cohort was approved by the Ethics Committee of Beijing Children's Hospital, Capital Medical University, National Center for Children's Health. Written informed consent to participate in this study was provided by the participants' legal guardian/next of kin.

## Author contributions

CH and WZ designed the study, analyzed the data, and revised the paper. RG analyzed the data. XZ, HL, and ZY recruited patients to this study and provided clinical data. HL wrote the manuscript. XZ supervised the study. All authors contributed to the article and approved the submitted version.

## Funding

This work has been supported by Beijing Hospitals Authority Youth Programme (QML20211204) and Beijing Talents Fund (2018000021469G275).

## Conflict of interest

The authors declare that the research was conducted in the absence of any commercial or financial relationships that could be construed as a potential conflict of interest.

## Publisher's note

All claims expressed in this article are solely those of the authors and do not necessarily represent those of their affiliated organizations, or those of the publisher, the editors and the reviewers. Any product that may be evaluated in this article, or claim that may be made by its manufacturer, is not guaranteed or endorsed by the publisher.
