# Supplementary material for: Genotypes and clinical intervention of patients with neurofibromatosis type 1 associated dystrophic scoliosis
Source: Front Pediatr. 2022 Aug 18;10:918136. doi: 10.3389/fped.2022.918136 (PMC9434403; doi:10.3389/fped.2022.918136)
Supplement: Supplementary file 1 [file Data_Sheet_1.PDF]

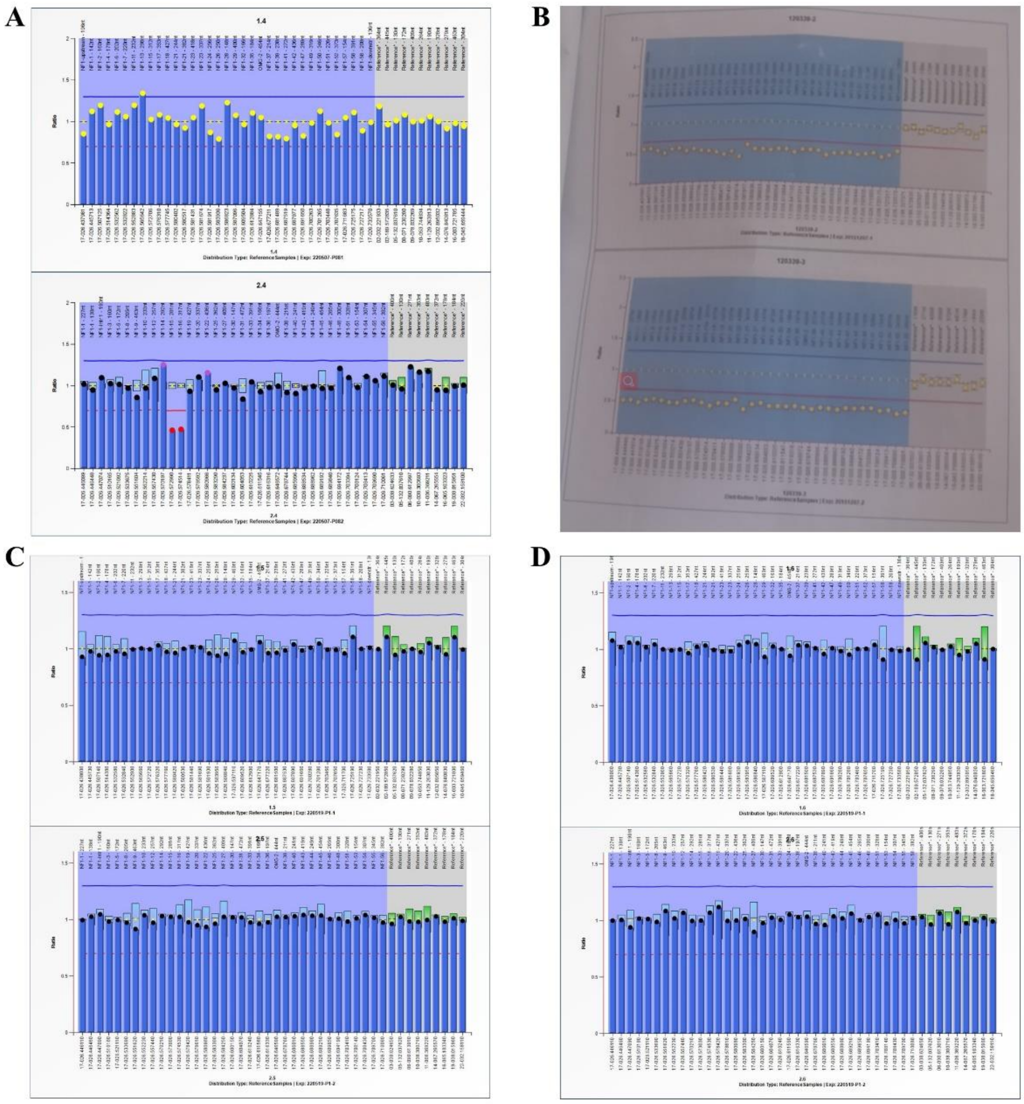

Supplementary Figure 1. The MLPA results of patients. **(A)** Patient 11 had deletion of 15th and 16th exons. **(B)** Patient 12 had deletion of the whole *NF1* gene. Patient 12 accepted ES and MLPA in other medical institution, and only the picture of MLPA results were offered. **(C)** Patient 13 had no exon deletion of *NF1*. **(D)** Patient 14 had no exon deletion of *NF1*.

Supplementary Table 1. The pathogenicity evaluation of novel *NF1* variants in patients

Patient ID	Nucleotide change	Amino acid change	Allele frequency				In-silico analysis	Classification
			ExAC_all	gnomAD_all	1000 Genomes	dbSNP		
5	c.1828delT	p.Leu611fs*19	0	0	0	0	-	P
6	c.2409_2409+1insGT	-	0	0	0	0	D	P
8	c.5247delA	p.Val1751fs*1	0	0	0	0	-	P
9	c.7095dupT	p.Asn2366*	0	0	0	0	-	P
10	c.5752_5756delATTGA	p.Leu1920fs*20	0	0	0	0	-	P

In-silico analysis for splicing mutation was predicted by GeneSplicer software (http://www.cbcb.umd.edu/software/GeneSplicer/gene_spl.shtml), D, deleterious. ExAC_all, the Exome Aggregation Consortium, all population; gnomAD_all, the Genome Aggregation Database, all population (http://gnomad.broadinstitute.org/); 1000 Genomes, The 1000 Genomes Project (https://www.internationalgenome.org/); dbSNP, database of single nucleotide Polymorphism (https://www.ncbi.nlm.nih.gov/snp/); P, pathogenic.
